# Tunisian revolution and the demand of healthcare in psychiatry outward department

**DOI:** 10.1192/j.eurpsy.2021.1880

**Published:** 2021-08-13

**Authors:** M. Regaya, B. Ben Mohamed, F. Zaafrane, B. Amamou, L. Gaha

**Affiliations:** Department Of Psychiatry, University of Monastir, Faculty of Medicine of Monastir, LR05ES10, Fattouma Bourguiba Hospital, Monastir, Tunisia

**Keywords:** mental health, environment, revolution

## Abstract

**Introduction:**

Tunisian revolution has been a major upheavel in the tunisian history and has brought many political, social and economic changes. Little were found about the revolution’s potential impact on the psychiatric demand.

**Objectives:**

Compare the clinical profile of all the new consultants in the out ward psychiatry department before and after the revolution.

**Methods:**

The study had a retrospective descriptive design including all the new consultants in the outpatient psychiatry department in the general hospital Fattouma Bourguiba in Monastir, Tunisia before (during 2007) and after (during 2016) the revolution. We used a pre-established questionnaire including sociodemographic and clinical data.

**Results:**

After the revolution, an increase in the number of new patients (p<10^-3^) 438 to 451 were found. In 2016, there were more unemployed consultants(p=0.004), having criminal record (p=0.01) and having a problematic substance use (p<10^-3^). An increase also concerned patients consulting for anxiety(p=0.002) and suicidal ideation (p=0.022). Considering the clinical diagnosis, there were also a significant increase regarding anxiety disorders (p=0.001) and mood disorders (p=0.011) essentially major depressive disorder (p=0.002). Although a significant decrease concerned somatoform disorder (p<10^-3^).

**Conclusions:**

Our study showed a change in the profile of consultants after the Tunisian revolution. A study in the general population could find specific etiological factors. Thus highlight the importance of implementing preventive measures in general population in crisis’ times.

**Disclosure:**

No significant relationships.

